# Experimental study on the transporting and crushing effect of gas on coal powder during the develop stage of coal and gas outburst in roadway

**DOI:** 10.1038/s41598-023-46023-0

**Published:** 2023-11-01

**Authors:** Jie Cao, Qianting Hu, Linchao Dai, Xuelin Yang

**Affiliations:** 1 School of Resources and Safety Engineering, Chongqing University, Chongqing, 400044 China; 2 State Key Laboratory of the Gas Disaster Detecting, Preventing and Emergency Controlling, Chongqing, 400037 China; 3 China Coal Technology and Engineering Group Chongqing Research Institute, Chongqing, 400037 China; 4 School of Safety and Emergency Management Engineering, Taiyuan University of Technology, Taiyuan, 030024 China

**Keywords:** Natural hazards, Engineering

## Abstract

In recent years, coal and gas outburst disasters are still occurring and difficult to prevent, seriously endangering the safety of coal mine production. It is well known that the transporting and crushing of outburst coal is the main pathway of energy dissipation during the coal and gas outburst process. However, a consensus regarding how much gas involves in outburst and affects energy dissipation is still lacking. Quantitative study on the gas effect on migration and fragmentation characteristics of outburst coal in restricted roadway space can improve the energy model and guide the prevention and control of gas outburst. In this paper, an improved visual coal and gas outburst dynamic effect simulation experiment system was used to conduct outburst simulation experiments at different gas pressure conditions. The results showed that the movement of outburst coal in the roadway has experienced various flow patterns. In the initial stage of the outburst, under low gas pressure condition, the motion of the outburst coal was dominated by stratified flow. However, as the gas pressure increases, the initial acceleration increases, and the outburst coal mainly move forward rapidly in the form of plug flow. The average velocity at 0.3, 0.5, and 0.8 MPa gas pressure condition were 6.75, 22.22 and 35.81 m/s, respectively. Gas also has a crushing effect on outburst coal. With increasing gas pressure, the number of coal powder particles of the same mass increased significantly, and the range of the particle size distribution of the particles decreaed, and the median particle size decreased. As the gas pressure increases, the outburst intensity gradually increases, and the total energy involved in the outburst work also increases. However, the energy dissipation pathways are different. At 0.3 MPa, the energy dissipation is dominated by crushing energy, which is about six times the ejection energy. As the gas pressure increased to 0.8 MPa, the proportion of the ejection energy gradually increases to about twice that of the crushing energy. Under the experimental conditions, 2.71–13.43% of the adsorbed gas involves in the outburst (AGIO) through rapid desorption, and the proportion increases with increasing gas pressure. This paper improves the energy model of coal and gas outburst, which is applicable to risk assessment and prevention of outburst disasters.

## Introduction

China's energy structure determines that coal makes a significant contribution to economic development^1,2^. At present, under the carbon neutrality target, coal is still the cornerstone of China's energy, and the energy transformation cannot be separated from the strong support of coal. In 2021, China produced about 4.13 billion tons of raw coal, more than half of the world's total output, accounting for 56% of China's primary energy consumption. The geodetic structure determines that the geological conditions of China's coal fields are complex, and underground mining is the main coal mining method. Coal and gas outbursts (CGO) have always threatened the safe production of coal mines in China^3–7^. In 2021, six CGO accidents happened in coal mines in China, killing 24 people, with year-on-year increases of 200% and 60%, respectively.

During outburst process, high-pressure gas engulfs the fractured coal body and quickly rushes from outburst hole to mining space or roadway, causing serious casualties and damage to the mining facility. The main outburst hazards are gas suffocation, coal powder burial, and coal–gas two-phase flow impact^8^. In the aftermath, an expert investigation team usually conducts inverse analysis of the outburst intensity and the causative conditions based on the roadway damage, ventilation facility damage, outburst coal ejection distance and accumulation, outburst coal powder particle size, and sensor record data at the outburst site. That is, the parameters of the disaster scene are used to deduce the disaster process, determine the causes of the accident, and provide guidance for the next step in disaster prevention and control.

At present, such deductions are mainly based on theoretical results. The energy relationship model of coal and gas outburst can satisfactorily explain the relationships between the geo-stress, gas, physical and mechanical properties of the coal or rock and outburst. It assumes that the accumulated energy of the outburst is mainly composed of elastic energy and the gas internal energy, and the gas internal energy is usually two to three orders of magnitude greater than the elastic energy^9,10^. However, there is no consensus by researchers regarding how much gas involves in the outburst (GIO). Wen et al. pointed out that only part of the free gas flowing from the pore spaces into the fractures involved in the outburst energy^11^. Experimental studies have shown that the amount of free gas alone cannot provide all the energy for coal rejection or crushing. Hu and Wen believed that some adsorbed gas also participated in outburst work through desorption^9^. Tu et al. studied the relationship between the amount of gas involving in the outburst and the particle size and diffusion coefficient of coal^12^.

To explore the mechanisms by which gas and the other main controlling factors influence on CGO, scholars conducted a series of one-dimensional and three-dimensional coal and gas outburst simulation experiments and investigated the impacts of various factors on the outburst intensity^13–18^. The multi-physical field coupling evolution law of stress, air pressure and temperature in the process of outburst occurrence was obtained through large-scale similarity simulation experiments^19–25^. However, the outburst ports in such experimental devices are all designed to be open, and the outburst coal powder ejects directly into the open space, ignoring the friction and collision between the coal powder particles in the confined space of the roadway. And as a result, these experiments do not simulate the actual coal powder migration processes. Whether the current research conclusions are universal still needs to be further explored^26^.

The ejection and crushing of outburst coal is the main pathway of energy dissipation during the outburst process. In recent years, more and more coal and gas outburst simulation devices are developed to simulated roadways, providing effective means to study the influence of gas on outburst coal powder migration and distribution. Chen first developed a set of CGO test systems with roadway^27^. So far, the study on the outburst shock wave induced by CGO has begun^28,29^. Since the entire roadway is welded with steel pipes, it is impossible to observe the migration process of coal and gas flow. The test systems developed by Zhou et al. included observation windows in local roadways to allow for observation of the two-phase flow in the roadways^30^. Using these devices, Sun et al. observed that the heights of the coal powder accumulation decreased with increasing distance from the outburst port in the roadway after outburst^31^. Xu et al. conducted systematic research on the impact dynamics of coal and gas two-phase flow in L-shaped roadways, and the mass of the outburst coal powder exhibited an increase–decrease–increase–decrease distribution pattern along the roadway and piled up in large quantities at the front of the right-angle corner^32,33^. Zhang et al. obtained a linear relationship between the crushing rate and relative outburst intensity, and the slope of the fitted line can be used to characterize the hazard posed by the outburst^34^. The simulation test systems made of transparent materials has been developed successively, allowing for visual observation of the two-phase flow in the roadways^35,36^. A preliminary study conducted by Liu et al. revealed that the ejected coal accumulated in roadway approximately exhibits a normal distribution, and the maximum flow rate of the coal particles observed 3.6 m from the outburst port is 25 m/s^37^. Jin et al. concluded that the coal powder flow in the roadway is mainly composed of four types of flow patterns: suspension flow, stratified flow, dune flow, and plug flow. However, the length of the roadway was designed to be short during their experiments, which may influence the development of coal and gas flow^38^.

From the point of view of energy, the outburst process is actual a process of energy accumulation and release. The ejection and crushing of the outburst coal is the main pathway of energy dissipation in this process^39–41^. Therefore, it is essential to make a quantitative analysis of the outbursts mechanism by studying the motion process and crushing characteristics of coal powder under the effect of gas, especially in the restricted space of the roadway. In this study, an improved visualized coal and gas outburst simulation test system was used to conduct outburst simulations under different gas pressure conditions. Based on the experimental results, the migration form, movement speed, mass and particle size distributions of outburst coal in the visualized restricted space of the roadways is analyzed. And the amount of gas involving in the outburst (GIO) from the perspective of energy can be investigated, which provide a basis for better comprehension of the outburst mechanism.

## Outburst coal migration and crushing experiments under the action of gas

### Experimental device

**Figure 1:**
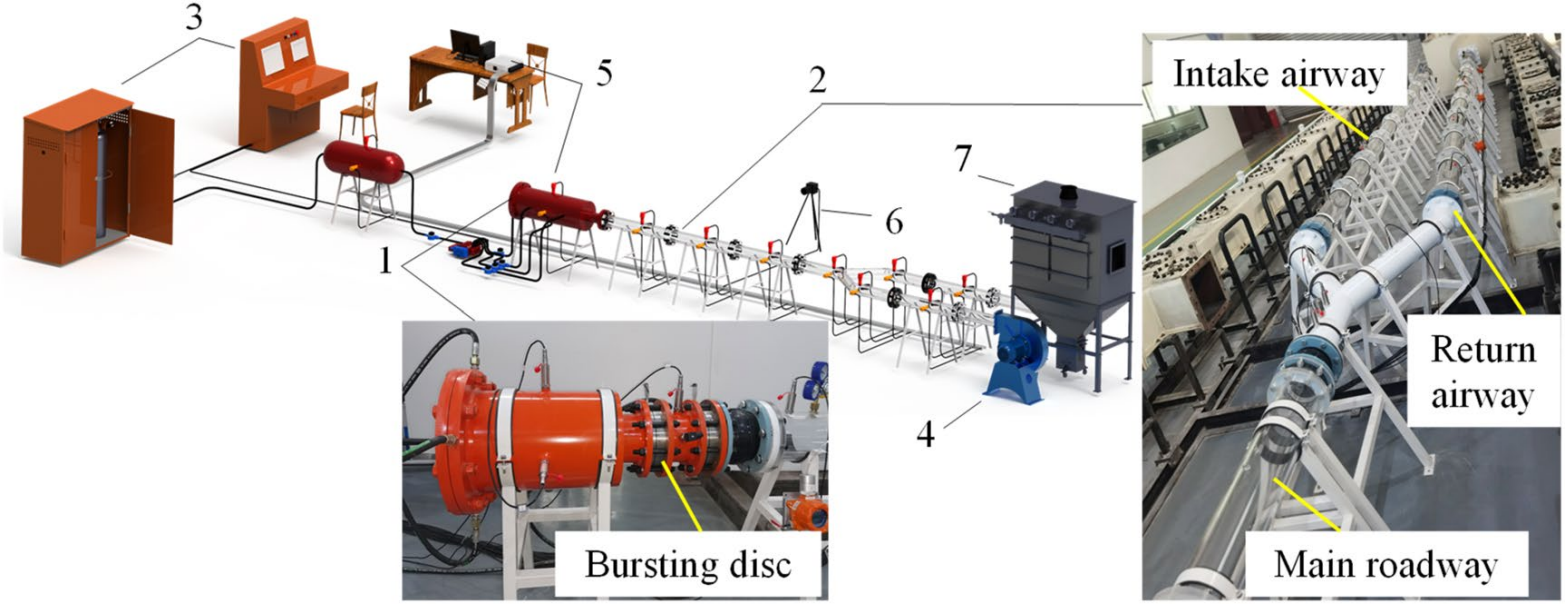
The visualization simulation test device for dynamic effect of coal and gas outburst. (1) Dynamic effect container; (2) visual roadway sub-system; (3) automatic inflation sub-system; (4) model fan; (5) data monitoring and collection devices; (6) high-speed camera; (7) dust removal sub-system.

To quantitatively analyze the mechanism of gas acts on outburst coal ejecting and crushing in a restricted roadway space during the CGO process, the original CGO dynamic effect simulation experiment system was improved (Fig. 1)^42,43^.

The outburst dynamic effect container consisted of a sealed high-pressure chamber and a rupture disc connector. The high-pressure sealed chamber was constructed of a cylinder of 304 stainless steel, with an inner diameter of 30 cm, a length of 45 cm, and a designed pressure resistance of 4 MPa. The back of the chamber was inflatable, which was achieved by using an inflatable flange. The flange contained four gas inlets on the outer edge of the flange, and the gas outlets were arranged evenly inside. This structure could realize surface inflation, which made the inflation uniform and efficient. The diameter of outburst mouth is set as 15 cm. And the bursting disc excitation method was adopted, with an opening time of about 20 ms, as shown in Table 1.

**Table 1:** Main parameters of the device and the existing ones.

Technical parameter	Outburst cavity size/cm	Outburst opening mode	Simulated pipe type	Simulated pipe size/cm
This device	φ30 × 45	Rupture disk	Acrylic material	φ15
Cao et al.^23^	φ100 × 236	Rupture disk	Metal, viewing window	30 × 30
Zhou et al.^30^	105 × 41 × 41	Rupture disk	Metal, viewing window	40 × 40
Hu and Wen^9^	φ30 × 45	Rupture of membranes	Plexiglass	20 × 20, arch radius 10
Zhao et al.^8^	φ20 × 30	Solenoid valve	Acrylic material	φ10
Chen^27^	φ30 × 45	Mechanical baffle	Metal	φ20
Jin et al.^38^	φ20 × 30	Mechanical baffle	Acrylic material	φ10
Wang et al.^36^	φ20 × 30	Crank slider	Acrylic material	φ10
Liu et al.^37^	φ20 × 50	Mechanical baffle	Acrylic material	φ20

The visual roadway sub-system was constructed of acrylic material with a high light transmittance and high strength. The inner diameter, the wall thickness, and the length of each pipe were 15 cm, the 2 cm, and 1 m, respectively. The pipes were connected and sealed with flanges and gaskets, and the total length was 50 m. According to the needs of the test, different types of sensor interfaces were reserved in different positions on the simulated roadway. Fans could be installed at the end of the roadway to simulate air supply or connected to the dust removal system to prevent the spread of harmful gases and coal dust.

### Experimental conditions and procedures

The coal samples used in the experiments were collected from the K_1_ coal seam of the 3114 south fully mechanized mining face in the Longtan Coal Mine, Sichuan Province. The K_1_ coal seam in the Longtan Coal Mine is an outburst coal seam. The gas content of the coal seam is 5.09–14.35 m^3^/t, with an average of 9.55 m^3^/t. The measured maximum gas pressure in the coal seam was 1.08 MPa. A total of 15 CGO accidents have occurred. The basic physical and mechanical parameters of the experimental coal samples are shown in Table 2.

**Table 2:** Coal sample parameters.

Parameters	TRD (g/cm^3^)	Porosity (%)	Proximate analysis			Adsorption constant		ΔP (mmHg)	*f*-value
			Mad (%)	Aad (%)	Vdaf (%)	a_CO2_ (cm^3^/g r)	b_CO2_ (MPa^−1^)		
Value	1.68	4.76	0.69	33.25	22.96	27.734	4.074	11	0.37

**Figure 2:**
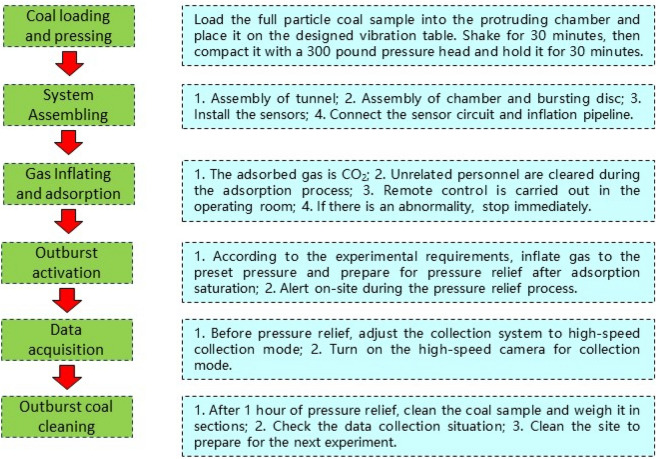
The experimental procedure flowchart.

To explore the migration and crushing characteristics of the outburst coal, outburst simulation experiments under different gas pressures (0.3 MPa, 0.5 MPa, and 0.8 MPa) were carried out using carbon dioxide as the experimental gas. The specific experimental steps is shown in Fig. 2. The length of the main roadway was designed to be 14 m in the experiment. Two 6-m-long branch roadways related to a 60° bifurcated roadway to form a Y-shaped roadway layout. The total weight of the loaded coal was 51 kg, and the voyage was 9.59%. A high-speed camera was used to capture real-time pictures of the migration of the coal powder in the first section of the roadway. Gas pressure sensors were installed on the outburst chamber and the roadway to monitor the pressure changes during the outburst process. After the experiment was completed, the coal samples ejected into the roadway were collected and statistically screened.

## Experimental results

### Variation law of gas pressure in outburst hole

Gas plays a major role in the development stage of coal and gas outburst. From the perspective of energy, the outburst development process is as follows: the gas expansion energy in outburst hole is continuously converted into other forms of energy, including the initial energy of the outburst shock wave and the energy of the coal ejection and crushing. And the moment of pressure release is accompanied by desorption of the adsorbed gas, making the research more difficult^44^. Therefore, it is necessary to observe the gas pressure variation in outburst chamber through experiments.

**Figure 3:**
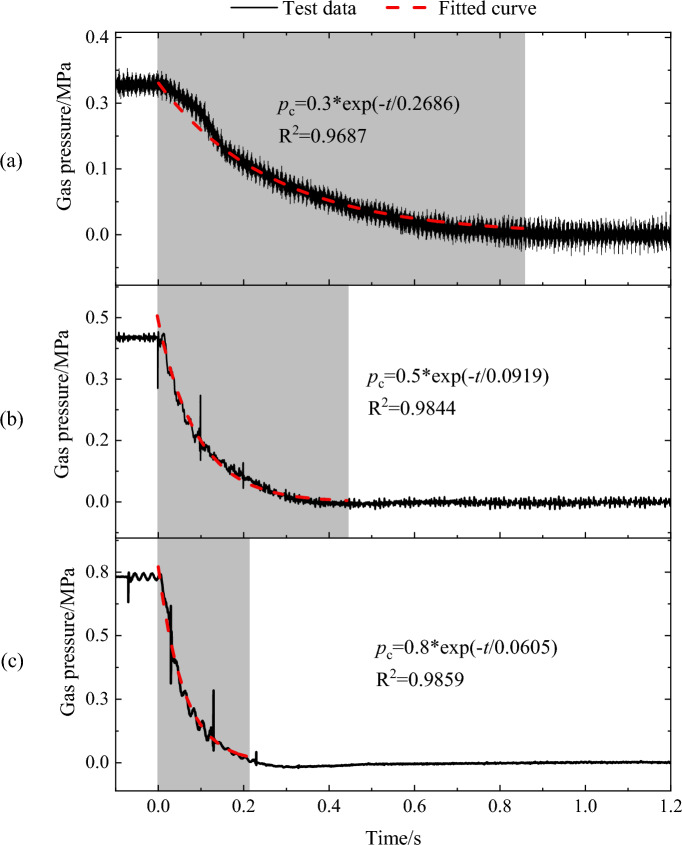
Variation law of gas pressure in outburst cavity under different gas pressures. (**a**) 0.3 MPa; (**b**) 0.5 MPa; (**c**) 0.8 MPa.

The variation of gas pressure in outburst cavity under different gas pressures is shown in Fig. 3. It was evident that as the gas pressure rose, the time for pressure to drop from the initial state to atmospheric pressure increasingly shortened, being 0.874 s, 0.441 s, and 0.236 s for pressures of 0.3, 0.5, and 0.8 MPa, respectively. The higher the gas pressure was, the greater the amount of gas released and the shorter the duration. As a matter of fact, during the outburst process, gas in the chamber will still be desorbed, with more gas being absorbed under higher gas pressure. It followed that the desorption rate of coal during the outburst procedure was lesser than the release rate of gas. Furthermore, a mathematical expression was represented by fitting the following function, as shown in Fig. 3, with all fitting coefficients surpassing 0.96, signifying that the equation can portray the law of gas pressure alteration in the outburst chamber.

1 $$ p_{c} = p_{0} \exp \left( { - \frac{t}{a}} \right) $$

where *p*_c_ is the pressure value in the outburst chamber at time *t*, MPa; *p*_0_ is the gas pressure in the chamber when outburst occurred, MPa; *t* is the time, s; and *a* is the fitting coefficient.

### Migration law of outburst coal–gas two-phase flow

During the coal and gas outburst, the gas mixed with broken coal powder sprayed out simultaneously in the roadway space, forming a high-speed gas–solid two-phase flow. Because the gas pressure released during the outburst is unsteady, the movement of the outburst coal–gas two-phase flow in the roadway space is also an extremely complex process. In order to study the kinetic characteristics of the interactions between the coal powder, gas, and air in the roadway after the outburst, a high-speed camera was placed in front of the first section of the pipe at the outburst port, and the pictures in the video were extracted frame by frame to obtain the motion state of the outburst coal–gas two-phase flow in the pipe at different times.

**Figure 4:**
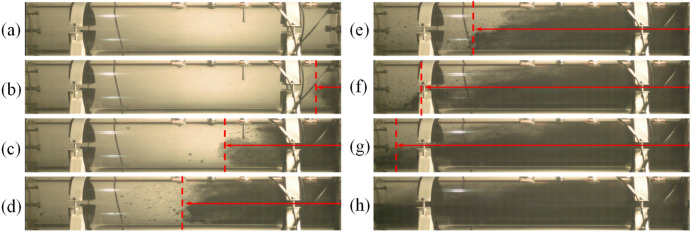
The evolution process of two-phase flow in the first section during outburst (0.3 MPa). (**a**) 0 ms; (**b**) 10 ms; (**c**) 50 ms; (**d**) 80 ms; (**e**) 100 ms; (**f**) 120 ms; (**g**) 140 ms; (**h**) 170 ms.

According to the experiment under 0.3 MPa condition, no coal powder was observed in the first section of the roadway 0.06 s after the outburst blasting sound (that is, the outburst occurrence). Taking the moment as the initial time, the coal powder was observed to be gradually ejected into the roadway space, as shown in Fig. 4. When the outburst occurred, the outburst coal powder was broken into different particle sizes. In the early stage of the outburst, due to the large amount of gas emitting out, the coal powder was ejected from the center of the outburst port, and continuously propelled forward under the action of the drag force of high-pressure gas (t = 10 ms). Meanwhile, due to the strong mobility of the gas flow, the speed of the coal powder increased rapidly, and a suspension flow was formed in the front section (t = 50 ms). As the coal powder flow moved forward, the carrying effect of the gas flow weakened, and the large coal particles behind the suspension flow gradually sank under the force of gravity, initially forming dune flow and soon after evolving into stratified flow (t = 100 ms). In the later stage of the outburst, the gas flow could no longer propel a large amount of coal powder. However, at this time, there were still continuous adsorbed gases released, carrying small coal powder particles in low-speed suspension movement, and filling the entire roadway (t = 170 ms). Then, as the gas pressure in the chamber continued to decrease, the gas emitting out was insufficient to continue throwing the coal, and the coal powder moving in the pipe gradually decelerated and settled due to friction and gravity.

**Figure 5:**
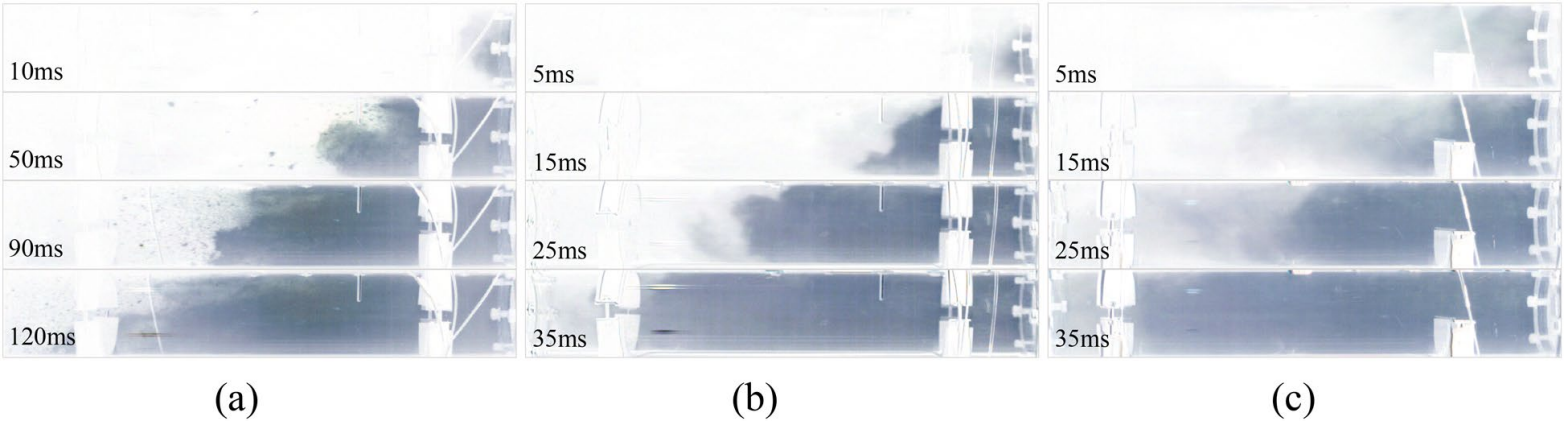
Grayscale image of coal outburst migration under different gas pressures. (**a**) 0.3 MPa; (**b**) 0.5 MPa; (**c**) 0.8 MPa.

In order to observe the movement process of the outburst coal powder, the pictures extracted from the high-speed camera video were compared and processed in gray scale using MATLAB. The migration diagrams of the outburst coal powder under different gas pressure conditions are shown in Fig. 5. The migration characteristics of the coal powder were different at different gas pressures. At 0.3 MPa, there was a large number of suspended coal powder particles in front, and the outburst coal gradually formed stratified flow after 90 ms, moving forward in a wedge shape overall. As the gas pressure increased, the number of suspended coal particles decreased. At 0.5 MPa, the ejected outburst coal formed dune flow within 15 ms, and then, it quickly formed plug flow (25 ms), moving forward and filling the roadway. However, at 0.8 MPa, a large amount of ultra-fine coal powder was expected to be ejected first. Then, the coal powder moved forward rapidly via plug flow driven by gas flow, and the first section of the pipe was filled in about 35 ms. It indicates that the greater the gas pressure is, the greater the expansion energy of the gas is, and the more obvious the crushing effect of the outburst coal powder is, and it can effectively overcome the gravity and resistance to form plug flow.

**Figure 6:**
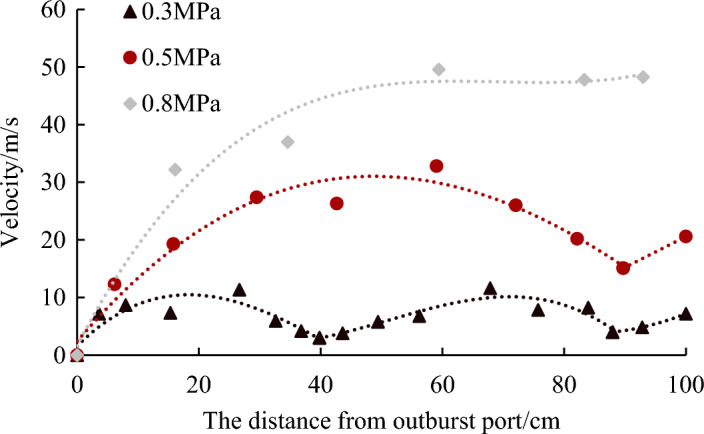
Variation of migration velocity of outburst coal–gas two-phase flow front under different pressures.

From the above analysis, the migration process of the outburst coal powder in the roadway underwent various forms of transformation. With the continuous desorption of the gas, the coal powder migration was difficult to maintain a uniform speed. The variation of ejected coal velocity under different gas pressures is shown in Fig. 6. At 0.3 MPa, the outburst coal powder accelerated gradually after injection, reached the first peak of 11.35 m/s at 26.65 cm, and then decelerated to 3 m/s at 39.8 cm. Then, it accelerated again, with a peak of 11.65 m/s, and then slows down again. Under this pressure condition, two acceleration-deceleration processes occurred within distance of 1 m. At 0.5 MPa, within 1 m, there was only an acceleration-deceleration process, with a maximum velocity of 32.8 m/s. At 0.8 MPa, only acceleration occurred. As the gas pressure increased, the average velocity of the migration of the coal powder within 1 m of the outburst port increased, which were 6.75, 22.22, and 35.81 m/s at 0.3, 0.5, and 0.8 MPa, respectively. Therefore, the distance of corresponding single acceleration-deceleration process gradually increased. In fact, according to the Bernoulli equation, the higher the gas pressure, the greater the initial velocity of the coal–gas two-phase flow.

### Mass and particle size distribution of the outburst coal powder

**Figure 7:**
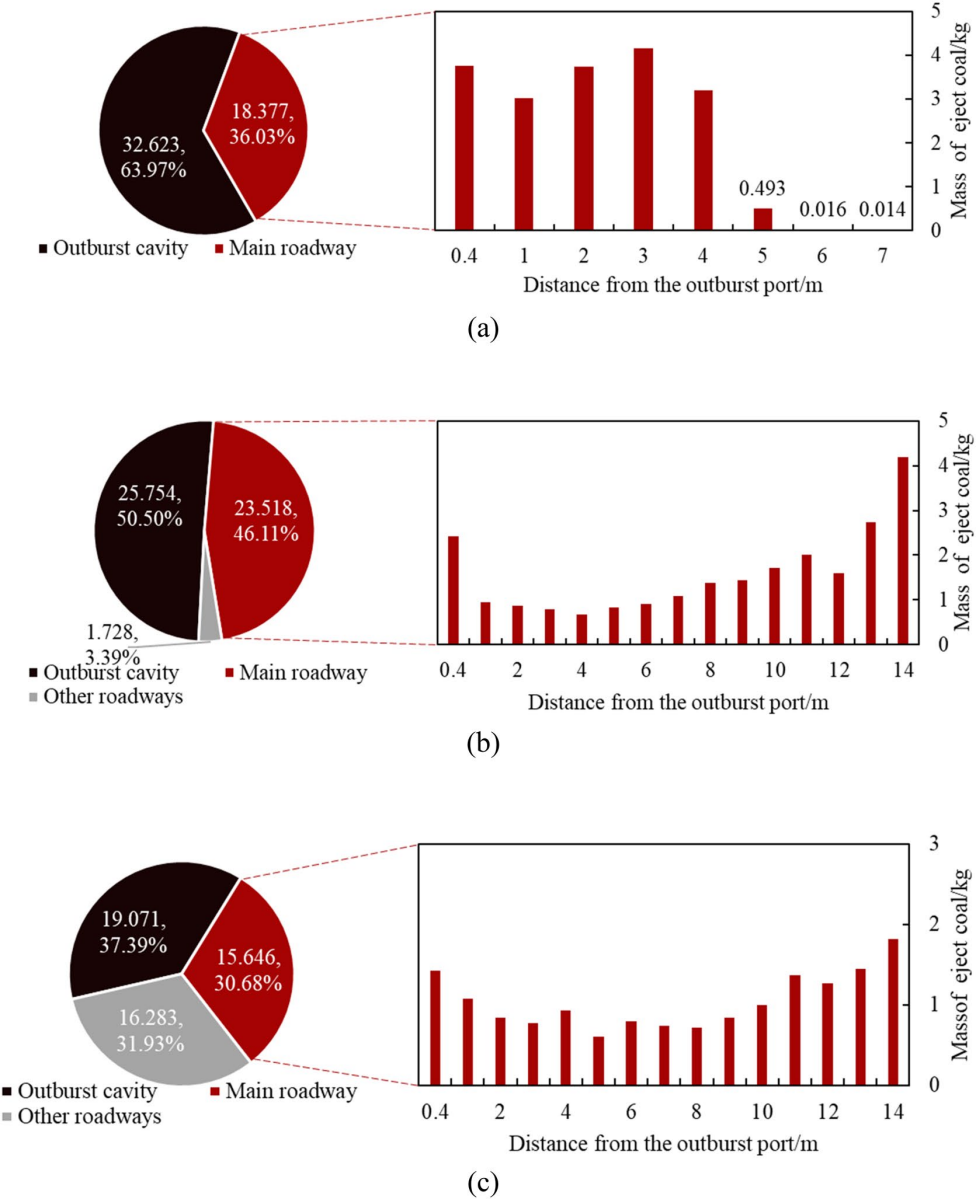
Mass distribution characteristics of outburst pulverized coal under different gas pressures. (**a**) 0.3 MPa; (**b**) 0.5 MPa; (**c**) 0.8 MPa.

The mass of the outburst coal powder is an important metric for measuring the outburst intensity. The mass distribution of the outburst coal powder at different gas pressures is shown in Fig. 7. At 0.3 MPa, the mass of the outburst coal was 18.377 kg, and the relative outburst intensity was 36.03%^45^. Most of them were distributed in the main roadway within 5 m of the outburst port, and it decreased with increasing distance from the outburst port. At 0.5 MPa, the relative outburst intensity increased to 49.5%, with 93.2% of the outburst coal powder being distributed in the main roadway, exhibiting the characteristics of heavy at both ends and light in the middle. This is due to that the coal ejection had a relatively high initial velocity under this experimental condition, and it maintained a certain rate until it reached to the junction. The movement of the coal body was stopped by obstacles, so there was more coal accumulation at the terminal junction of the main road. When the gas pressure increased from 0.5 to 0.8 MPa, the relative outburst intensity increased to 62.61%, an increase of 26.5%. It indicates that as the gas pressure increased, the outburst intensity gradually increased, with a decreasing rate of increase. In addition, at 0.8 MPa, due to the greater ejection velocity, the mass of the coal powder in the branch roadway accounted for 51% of the outburst coal mass.

**Figure 8:**
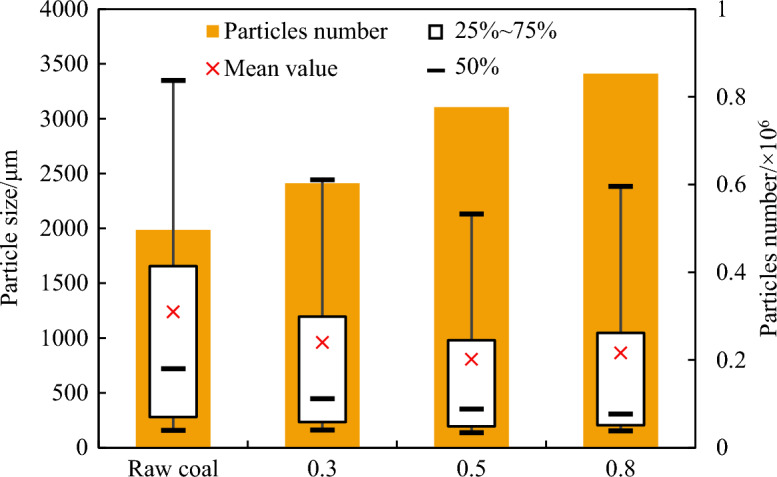
Box diagram of different particle size distributions.

In the process of outburst development, coal powder particles are continuously crushed into smaller particles because of the gas pressure gradients inside and outside of the surface, as well as the collision and friction between particles. The crushing effect of outburst coal bodies can not only be observed in experiments but has also been confirmed in many outburst case studies. To analyze the crushing effect, the outburst coal powder was collected, and the particle size was analyzed using an OCCHIO ZEPHYR ESR2 particle size analyzer, which has a wide measurement range of 30 µm to 30 mm. The measurement steps were as follows: The coal powder after outburst in each section of roadway was evenly mixed. Then, 200–300 g of coal powder was randomly screened for measurement. If the mass in a single section of roadway was less than 200 g, all measurements would be made. The box diagrams and the number of the particle size distribution of the coal powder are presented in Fig. 8. Each node of the box diagram represents the corresponding particle size for cumulative masses of 10%, 25%, 50%, 75%, and 90%. The original coal sample particles were primarily distributed between 157 µm and 3349 µm, with an average particle size of 1239 µm and a median particle size of 719 µm. The outburst coal samples were primarily distributed between 135 and 2442 µm due to the crushing effect, and as the gas pressure increased, the median particle size decreased, with sizes of 446 µm, 352 µm, and 307 µm at 0.3, 0.5, and 0.8 MPa, respectively. Due to the original coal sample was broken up during outburst, the particle number corresponding to the same mass increased. The greater the gas pressure was, the more significant the crushing effect was. The number of particles gradually increased from the original 0.5 million to 0.85 million.

## Discussion

### Factors affecting the migration velocity of the outburst coal powder

The transport of outburst coal is one of the main forms of the gas internal energy dissipation in coal seams. Through experimental observations, the outburst coal particles experience various forms of transformation during their migration in the roadway, with a non-constant migration velocity, making the process relatively complicated. In previous studies^31^, the migration of the outburst coal powder particles was regarded as free suspension movement. To facilitate analysis, only the average velocity of the gas flow along the axis of the roadway was taken into consideration, ignoring its fluctuations, the fluctuation of the gas flow rate perpendicular to the axis of the roadway, and the radial component of the flow velocity of ejected coal particles. Based on the suspension movement mechanism of the solid particles in two phase flow and the law of energy conservation, a one-dimensional mathematical model of the migration of the outburst coal particles in the roadway was established. This model can be divided into a particle group acceleration stage and a gas–solid equilibrium movement deceleration stage.

2 $$ \begin{gathered} L{ = }L_{{\text{a}}} { + }L_{d} \hfill \\ L_{a} \left\{ \begin{gathered} \left( {\frac{v - u}{{v_{n} }}} \right)^{2 - K} - \frac{{v_{n} }}{v} - \frac{{\lambda_{s} u^{2} }}{2gD} = \frac{u}{{\text{g}}}\frac{du}{{dL}} \hfill \\ \left( {1 - \lambda_{g} \frac{\Delta L}{D}} \right)v_{c}^{2} + n\left( {1 - \lambda_{s} \frac{\Delta L}{D}} \right)u_{c}^{2} = v^{2} + nu^{2} \hfill \\ \end{gathered} \right. \hfill \\ L_{d} \left\{ \begin{gathered} \left( {\frac{v}{{v_{n} }}} \right)^{2 - K} \left[ {1 - \left( \frac{u}{v} \right)^{2 - K} } \right] - \frac{{v_{n} }}{v} - \frac{{\lambda_{s} u^{2} }}{2gD}\left( \frac{u}{v} \right)^{2} = 0 \hfill \\ \Delta L = \frac{{\left[ {\left( {v_{c}^{2} - v^{2} } \right) + n\left( {u_{c}^{2} - u^{2} } \right)} \right]D}}{{\lambda_{g} v_{c}^{2} + n\lambda_{s} u_{c}^{2} }} \hfill \\ \end{gathered} \right. \hfill \\ \end{gathered} $$

where *L* is the total migration distance of outburst coal, m; *L*_a_ is the migration distance of the outburst coal acceleration stage, m; *L*_d_ is the migration distance of deceleration stage, m; ∆*L* is the distance value of each calculation step, m; *u* and *v* are outburst coal particle and gas flow velocity, m/s; *u*_c_ and *v*_c_ are respectively the initial velocity of outburst coal and gas flow in the calculation stage, m/s; *v*_n_ is suspension velocity, m/s; *g* is the acceleration of gravity, N/kg; *D* is roadway diameter, m; *λ*_g_ is the pressure loss coefficient along the flow path; *λ*_s_ is the resistance coefficient of particles group; *n* is solid–gas mass ratio; the value of *K* is 0 or 1, when the diameter of outburst coal particles is large and Newton's resistance formula is obeyed, *K* = 0; when the diameter of coal particles is small and the Stokes resistance formula is obeyed, *K* = 1, Reynolds number can be used to evaluate the motion state of particles, as shown in Table 3.

**Table 3:** Particle size range and suspension velocity in different motion states^46^.

Motion state region	Particle size range	Suspension velocity
Newtonian resistance region 500 < Re ≤ 2000	$20.4\left[ {\frac{{\mu^{2} }}{{\rho \left( {\rho_{s} - \rho } \right)}}} \right]^{\frac{1}{3}} < d_{s} \le 1100\left[ {\frac{{\mu^{2} }}{{\rho \left( {\rho_{s} - \rho } \right)}}} \right]^{\frac{1}{3}}$	$\nu_{n} = 5.45\sqrt {\frac{{d_{s} \left( {\rho_{s} - \rho } \right)}}{\rho }}$
Stokes resistance zone Re ≤ 500	$2.2\left[ {\frac{{\mu^{2} }}{{\rho \left( {\rho_{s} - \rho } \right)}}} \right]^{\frac{1}{3}} < d_{s} \le 20.4\left[ {\frac{{\mu^{2} }}{{\rho \left( {\rho_{s} - \rho } \right)}}} \right]^{\frac{1}{3}}$	$\nu_{n} = 1.195d_{s} \left[ {\frac{{\left( {\rho_{s} - \rho } \right)^{2} }}{\mu \rho }} \right]^{\frac{1}{3}}$

Where *d*_s_ is the particle size of outburst coal, m; *μ* is the dynamic viscosity, Pa s; *ρ* and *ρ*_s_ are the density of gas and coal particle, kg/m^3^, respectively.

It is known that at the outburst port, *L* = 0 and the initial coal particle velocity *u*_*c*_ = 0; and at the end of the coal powder migration, the gas velocity *v* = *v*_n_. The initial velocity of the outburst gas flow can be calculated as follows^47^:

3 $$ u_{c} = \sqrt {\frac{2k}{{k - 1}}\frac{{p_{0} }}{\rho }\left[ {1 - \left( {\frac{{p_{c} }}{{p_{0} }}} \right)^{{\frac{k - 1}{k}}} } \right]} $$

where *k* is the thermodynamic coefficient of gas. The relationship between the total migration distance *L*, the maximum movement velocity *v*_max_ and the initial gas flow velocity *v*_c_ can be obtained by step calculation.

#### Relationship between migration velocity and time of outburst coal powder

According to Table 3, the calculated critical particle size of the Newtonian resistance zone and the Stokes resistance zone under the experimental conditions was 850 µm, and the corresponding suspension rate was 4.72 m/s. From the above experimental results, at the experimental gas pressures, about 60–68% of the outburst coal powder particles were smaller than the critical particle size.

**Figure 9:**
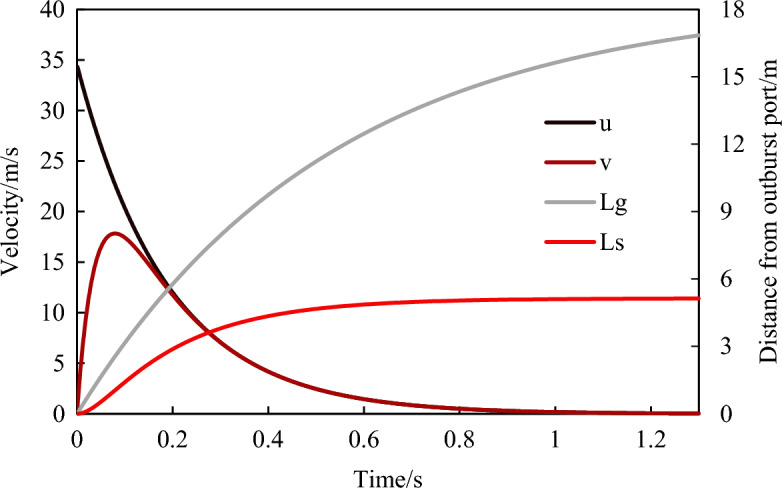
The relationship between coal powder migration velocity, distance and time.

Taking the migration of the 850 µm coal powder particles under gas pressure of 0.3 MPa as a calculation example, the relationship between the migration velocity, distance, and time of the outburst coal powder were obtained, as shown in Fig. 9. The gas velocity gradually decreased as the outburst developed, and the movement of the coal powder particles initially increased rapidly and then decreased. The migration velocity of the coal powder particles increased rapidly to a peak value of 17.84 m/s in a very short period (79 ms, corresponding to a migration distance of 1.04 m). It then gradually matched the gas velocity and decreased as the gas velocity decreased. When the particle suspension rate was reached, settling began. Under this condition, the migration distance of the coal powder particles was about 5.12 m, which was essentially consistent with the experimental results described in Section “Mass and particle size distribution of the outburst coal powder”.

#### Relationship between outburst coal migration velocity and gas pressure attenuation in outburst cavity

**Figure 10:**
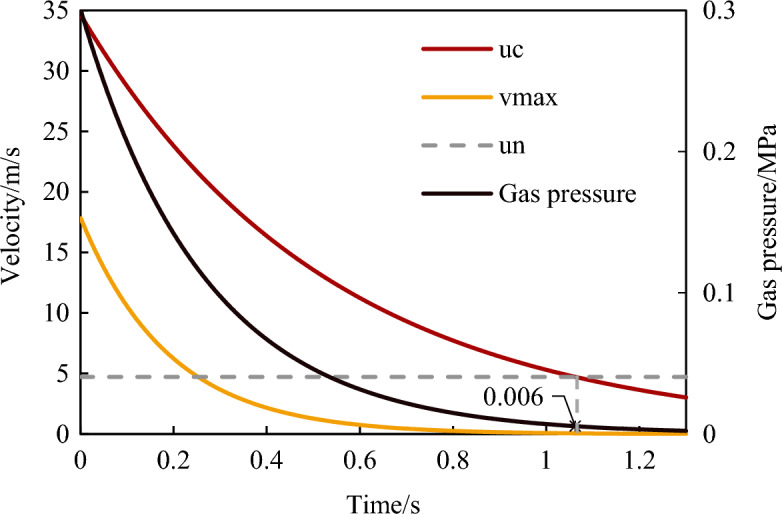
The evolution law of gas pressure, coal and gas migration velocity in the outburst development process.

According to Section “Variation law of gas pressure in outburst hole”, the gas pressure in the outburst chamber decreased as a negative exponential function with time, indicating that the initial rate *v*_c_ of the gas flow emitting out of the outburst port also changed. Using Eqs. (2) and (3), the relationship between the initial gas velocity, the maximum migration velocity of the outburst coal, and time could be calculated, as shown in Fig. 10. With the development of outburst, the initial gas flow velocity gradually decreased, and the maximum velocity and migration distance of the corresponding coal powder particles also gradually decreased. Moreover, when the gas pressure was lower than 0.006 MPa, the gas flow velocity did not reach the suspension velocity corresponding to this particle size, and the coal powder could not continue to eject forward. The gas pressure corresponding to the suspension velocity is defined as the critical pressure *p*_*l*_, and it is considered that when the gas pressure drops below the critical pressure, no work is done on the outburst. From the equations in Table 3, the smaller the corresponding suspension rate is. Therefore, as the outburst developed, the gas pressure in the chamber gradually decreased, and the equivalent particle sizes of the ejected-out coal powder particles gradually decreased. In the later stage of the outburst, only the coal powder with extremely small particle sizes was suspended at low velocity.

#### Effect of gas pressure fluctuation on migration velocity in roadway

**Figure 11:**
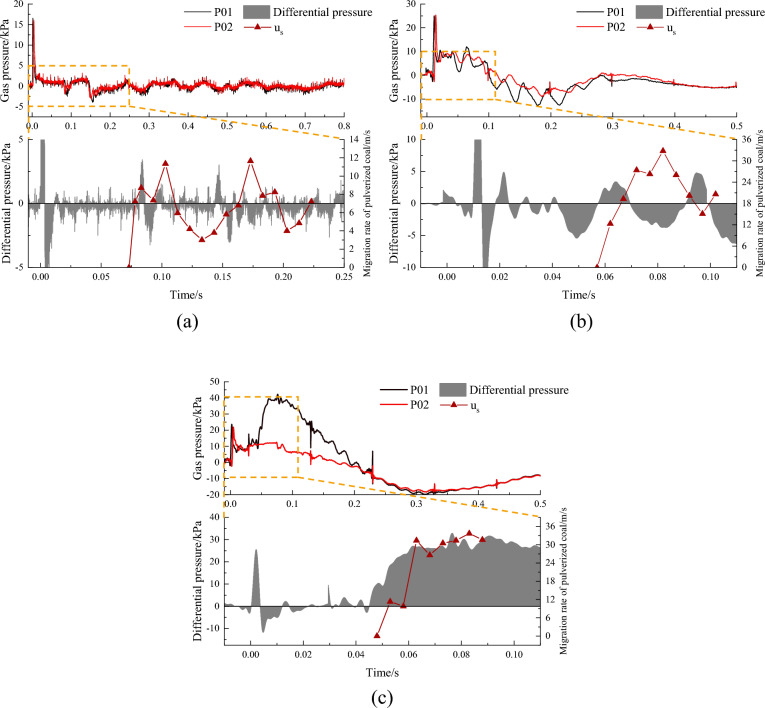
Relationship between airflow disturbance and coal powder migration velocity in roadway. (**a**) 0.3 MPa; (**b**) 0.5 MPa; (**c**) 0.8 MPa.

The above analyses can explain the overall migration process of the outburst coal powder particles based on the assumption of ignoring the fluctuations in the average velocity of the gas flow. When outburst occurs, a large amount of gas emits into the roadway space in a very short period, compresses the roadway air, and forms a shock wave^28,29,42,48^. During experimental process, accompanied by a loud bang, the overpressure at the measuring point of the pressure sensor arranged on the roadway increased sharply to the peak value. It maintained a certain positive pressure value and then decreased to normal pressure, and then it produced negative pressure due to the over-expansion of the gas as it passed the measuring point. Figure 11 shows the variation of gas pressure of P01 and P02 arranged on the first and second sections of the roadway. With the increase of gas pressure, the overpressure peak value increased, over-expansion occurred, and thus, the negative pressure peak value increased. Therefore, due to the impact of the outburst impact gas flow disturbance, the gas pressure in the roadway was in a state of positive and negative pressure disturbances for a certain period. Under gas pressures of 0.3, 0.5, and 0.8 MPa, the high-speed camera showed that the outburst coal powder appeared at 0.073 s, 0.057 s, and 0.048 s, respectively. The variations in the difference pressure between P01 with P02 and the outburst coal powder migration rate with time are also shown in Fig. 11. After the outburst coal powder started to be ejected, the change in the velocity of the outburst coal powder was essentially consistent with the positive and negative fluctuations in the difference pressure in the first and second sections of the roadway. At 0.3 MPa and 0.5 MPa, the difference pressure during the observation period of the coal powder migration exhibited positive and negative fluctuations, and the velocity of coal powder migration was attenuated by the negative pressure. At 0.8 MPa, during the observation period of the coal powder migration, the pressure gradient reached 30 kPa and remained at this value for a period, providing a stable power source for the increase in the coal powder velocity. These results show that after the outburst occurred, the migration velocity of the outburst coal powder particles increased rapidly, but it was affected by the negative pressure disturbance in the roadway. The coal powder migration velocity calculated using Eq. (2) is greater than the actual value because the ignoring of the effect of the gas pressure fluctuation in the calculations. Therefore, it is of great significance to analyze the actual migration velocity of the outburst coal powder through experiments.

### Energy analysis of coal and gas outburst

The outburst process is a process of energy accumulation and release. The migration and crushing of the outburst coal is the main pathway of energy dissipation in this process. Considering that the outburst of coal and gas occurs instantaneously, the entire gas expansion process is very short, so it can be approximately considered to be an adiabatic process, and the following energy relationship model can be obtained:

4 $$ \left\{ \begin{aligned} & W = A_{1} + A_{2} \\ & W = V\frac{{p_{1} }}{n - 1}\left[ {\left( {\frac{{p_{0} }}{{p_{1} }}} \right)^{{\frac{n - 1}{n}}} - 1} \right] \\ & A_{1} = \alpha \cdot \Delta S,\;\Delta S = S_{T} - S_{0} = \frac{{6 \times 10^{4} G}}{\rho }\left( {\frac{1}{{d_{T} }} - \frac{1}{{d_{0} }}} \right) \\ & A_{2} = \frac{{m\nu^{2} }}{2} = \frac{{G\nu^{2} }}{2} \\ \end{aligned} \right. $$

where *W* is the energy accumulated by outburst, J, which is composed of coal elastic energy and gas internal energy. The experiment of this paper was only gas internal energy. *A*_1_ is the crushing energy, J; *A*_2_ is the transport energy, J; *V* is the volume of gas involved in outburst work, m^3^; *p*_1_ is the gas pressure after the outburst, MPa. According to the previous analysis, when the gas pressure drops to the critical gas pressure *p*_*l*_, the gas pressure in the hole will no longer work on the outburst, that is, *p*_1_ = *p*_*l*_; *n* is the adiabatic coefficient; *α* is the crushing specific work, J/cm^2^, that is the work dissipated by the new unit surface area; ∆*S* is the new surface area produced after crushing, cm^2^; *S*_0_ and *S*_*T*_ are the total surface area of coal samples before and after outburst, cm^2^; *G* is the mass ejected coal, kg; *d*_0_ and *d*_*T*_ are the mean diameters of coal before and after outburst, mm; *ν* is the migration velocity of ejected coal powder, m/s.

#### Dissipation of CGO energy

The outburst accumulated energy and the dissipated energy can be obtained from Eq. (4). The main undetermined parameters in the equation are the migration velocity of the outburst coal powder and the crushing specific work. From the analysis above, the migration velocity of outburst coal powder can reach its peak value in a very short time, and then gradually decrease. Moreover, as the outburst develops, the gas pressure in the chamber gradually decreases, and the average particle size and migration velocity peak value of the outburst coal powder gradually decrease. In addition, the migration velocity is also affected by the gas flow disturbance in the roadway. The migration process of the outburst coal powder is complex and has many influencing factors. Therefore, the mean velocity of ejected coal in the initial stage obtained from the experiments can be used as the calculated value. As for the studies of the crushing specific energy. Cai and Xiong^49^ used coal samples with different hardness to carry out drop hammer tests and obtained the relationship between the crushing specific energy and the firmness coefficient *f* of the coal, which has been adopted by some scholars^41,50^. The data from hammer drop tests in this paper conforms to this relationship.

**Figure 12:**
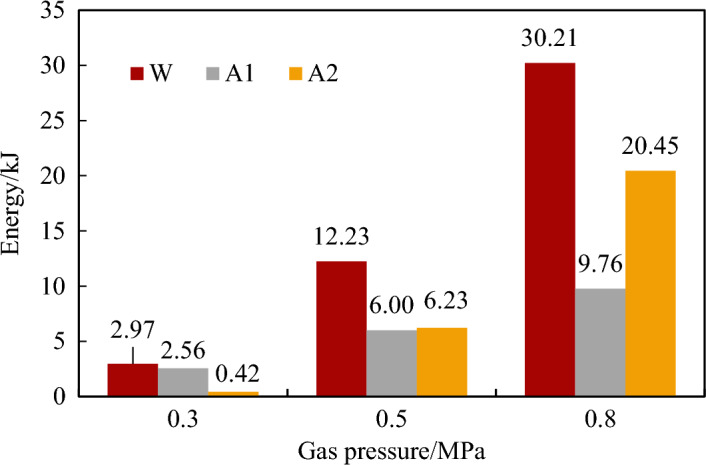
The accumulated and dissipated energy of outburst under different gas pressures.

Therefore, the outburst energy at different gas pressures can be obtained. From Fig. 12, with the increase of gas pressure, the total energy involved in the outburst work also increased. The main energy dissipated in different ways under different conditions. At 0.3 MPa, the energy dissipation was dominated by crushing energy, which was about 6 times the transport energy. At 0.5 MPa, the crushing energy was essentially the same as the transport energy. When the gas pressure increased to 0.8 MPa, the transport energy was about twice the energy consumed by the crushing. The reason for this is mainly that the gas pressure affects the transport of the coal powder from two aspects. First, the outburst gas pressure directly determines the initial velocity of the gas flow at the outburst port. The maximum gas flow velocities at 0.3, 0.5, and 0.8 MPa increase significantly, reaching 34.61 m/s, 77.98 m/s, and 144.64 m/s, respectively, resulting in increases in the maximum migration velocity of the outburst coal powder. Second, the increase in the gas pressure leads to an increase in the crushing effect on the coal powder and a decrease in the particle size of the outburst coal powder. According to the analysis presented in Section “Factors affecting the migration velocity of the outburst coal powder”, the corresponding suspension velocity decreases, and the critical gas pressure value decreases. These results indicate that the amount of internal gas energy that can be used for coal powder migration work in the outburst development process also increases. Therefore, with the increase in the gas pressure of outburst, both the crushing energy and the transport energy tend to increase, but the transport energy increases more obviously.

#### The gas amount involved in CGO

**Figure 13:**
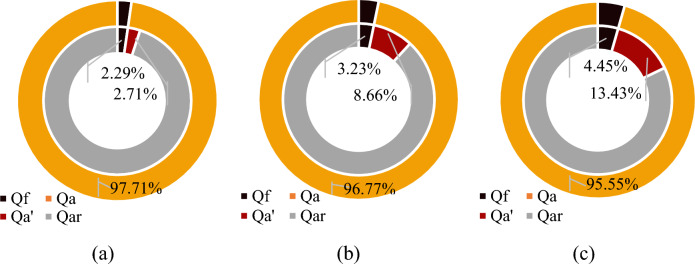
The proportions of the free gas and adsorbed gas of coal sample and the GIO. (**a**) 0.3 MPa; (**b**) 0.5 MPa; (**c**) 0.8 MPa.

The outburst energy all came from the internal gas energy in this paper. According to the calculation, the volume of gas involved in the outburst (GIO) were 0.031, 0.088, and 0.153 m^3^ at 0.3, 0.5, and 0.8 MPa, respectively. The volume of free gas in the sealed pressure chamber of the experiment were 0.014, 0.024, and 0.038 m^3^, respectively, which were less than the volume of GIO. Assuming free gas participated in the outburst work, it indicates that a large volume of adsorbed gas involved in the outburst (AGIO) through desorption and expansion. The total volume of gas in the coal sample and the proportions of the GIO are shown in Fig. 13. The outer circle in Fig. 13 represents the total gas content, which consists of the volume of free gas *Q*_f_ and the volume of adsorbed gas *Q*_a_. The inner circles represent the volume of GIO and the residual quantity of gas *Q*_ar_, where Qa' is the volume of AGIO. It can be concluded that under the experimental conditions, 2.71–13.43% of the adsorbed gas rapidly desorbed and participated in the outburst work.

**Figure 14:**
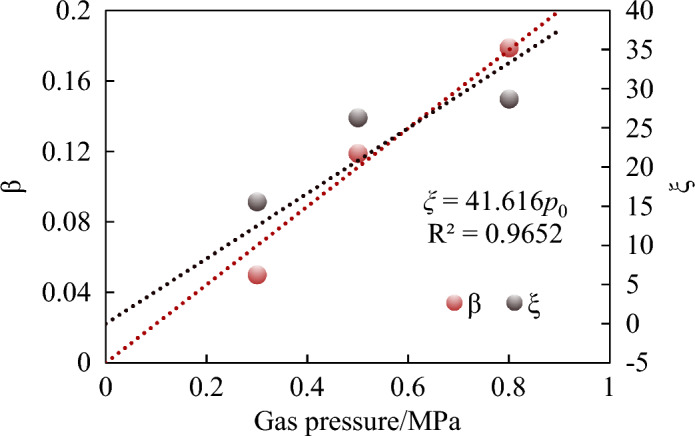
The proportional coefficient under different gas pressures.

The amount of GIO has always been a challenge for outburst energy analysis. The empirical formula proposed by scholars at present are shown in Table 4. At present, it has been confirmed by experiments that part of adsorbed gas participates in outburst work through rapid desorption. Tu et al. derived a formula of calculating the amount of GIO^12^. But many parameters in the Tu’s formula are difficult to obtain in the field. However, Hu and Wen did not give a clear explanation of the value of empirical ratio coefficient^9^. According to the analysis of the experimental results, the proportional coefficient of the amount of GIO to the gas content or the free gas is not constant, and its relationship with the gas pressure is shown in Fig. 14. The reason is that at different gas pressures, the crushing degree of the outburst coal is different^51^, and the migration law of the outburst coal powder is also different. As the gas pressure increases, the energy conversion efficiency increases and the crushing degree increases, which is manifested as a decrease in the particle size and an increase in the specific surface area after the outburst. The increase in the specific surface area, in turn, affects the increase in the amount of gas desorption, and the amount of GIO increases.

**Table 4:** The empirical formula of amount of GIO.

Formula	Format	Parameter
Hu and Wen^9^	$V = \xi \phi V_{s}$	Where* ϕ* is porosity of coal mass, %; *V*_*s*_ is the volume of the energy release zone of outburst, m^3^; *ξ* is the proportional coefficient, > 1.0, which indicates that in addition to free gas, part of the adsorbed gas becomes free gas through desorption, and also involves in the outburst work
Tu et al.^12^	$V = V^{f} + V^{a} = \phi V_{c} \left( {\frac{{p_{1} }}{{p_{0} }}} \right)^{\frac{1}{n}} + \frac{12}{{\sqrt \pi }}\left( {\frac{D}{{d^{2} }}t} \right)^{\frac{1}{2}} Q_{\infty }$	Where *V*^*f*^, *V*^*a*^ is volume of free and adsorbed gas involved in the outburst, m^3^; *V*_*c*_ is volume of outburst coal mass, m^3^; *D* is diffusion coefficient, m/s; *t* is outburst time, s; Q_∞_ is limiting volume of gas desorbed in coal particles, m^3^

Therefore, the outburst energy model expressed in Eq. (4) can be rewritten as follows:

5 $$ \chi p_{0} \phi V_{s} \frac{{p_{l} }}{n - 1}\left[ {\left( {\frac{{p_{0} }}{{p_{l} }}} \right)^{{\frac{n - 1}{n}}} - 1} \right] = \alpha \cdot \Delta S + \frac{{G\nu^{2} }}{2} $$

where *χ* is the fitting coefficient, and *χ* = 41.616 for the coal sample properties in this experiment. *p*_*l*_ is the critical gas pressure, MPa, it shows that when the gas pressure is lower than this value, the gas in the outburst hole no longer do work to the outburst.

This conclusion is useful for predicting and identifying the critical conditions for outburst occurrence. In fact, the crushing degree of the outburst coal is not only affected by the gas pressure but is also related to the firmness coefficient of the coal itself. Therefore, the quantity of gas participating in the outburst work is also related to the parameters such as the firmness coefficient of the coal. Under the same conditions, the lower firmness coefficient is, the higher crushing degree is, which is more conducive to the instantaneous release of gas internal energy to do work on the outburst. Therefore, relevant studies can be carried out in the future to further analyze the multi-factor coupled influence mechanism involved in the gas quantity participating in outburst work.

## Conclusions

To clarify the mechanism of gas acts on the outburst coal ejection and crushing in a restricted roadway space during the outburst development process, outburst simulation experiments at different gas pressure conditions was conducted by an improved visual CGO dynamic effect simulation experiment system. The law of coal powder ejection and crushing in restricted roadway space was obtained. The effect of gas pressure on ejected coal migration and the GIO was studied. The main conclusions are as follows:

1. The migration process of the outburst coal powder in simulated roadway under the action of gas underwent various forms of transformation. In the initial stage of the outburst, the movement form of the outburst coal powder was mainly stratified flow under low gas pressure condition. However, as the gas pressure increased, the coal powder moved forward rapidly in the form of plug flow. The higher the gas pressure was, the faster the coal powder migration velocity was. The initial average velocities were 6.75, 22.22, and 35.81 m/s at 0.3, 0.5, and 0.8 MPa, respectively.
2. As the gas pressure increased, the outburst intensity gradually increased, but the rate of increase decreased. The relative outburst intensities were 36.03%, 49.5%, and 62.61%. The greater the gas pressure was, the farther the coal powder was ejected. The proportions of the coal powder distributed in the branch roadway (distance > 14 m) under gas pressures of 0.3, 0.5, and 0.8 MPa were 0%, 3.39%, and 31.93%, respectively. The gas also had a crushing effect on the outburst coal bodies. As the gas pressure increased, the number of particles of the same mass increased significantly, the particle size distribution range decreased, and the median particle size decreased, with values of 446 µm, 352 µm, and 307 µm.
3. The change in the migration velocity of the outburst coal powder was mainly divided into an accelerated stage of the particle groups and a deceleration stage of the gas–solid equilibrium. The velocity in the acceleration stage was affected by the decrease in the gas pressure in the outburst holes and the fluctuation of the gas flow in the roadway. During the outburst process, the outburst hole pressure decreased as a negative exponential function of time, and the corresponding maximum velocity and migration distance of the coal powder ejected also gradually decreased. The gas pressure corresponding to the suspension rate is defined as the critical gas pressure. When the gas pressure drops below the critical gas pressure, it is unable to provide effective energy for the outburst.
4. As the gas pressure increased, the total energy of the outburst work increased. However, the energy dissipated in different ways. At 0.3 MPa, the energy dissipation was dominated by crushing energy, which was about six times that of the transport energy. As the gas pressure increased to 0.8 MPa, the proportion of the transport energy gradually increased to about twice that of the crushing energy. Under the experimental conditions, 2.71–13.43% of the adsorbed gas rapidly desorbed and participated in the outburst work. The proportional coefficient of the amount of GIO increased with increasing gas pressure.

## Data Availability

The data that support the findings of this study are available from the corresponding author upon reasonable request.
